# Using eye tracking and gaze pattern analysis to test a “dirty bomb” decision aid in a pilot RCT in urban adults with limited literacy

**DOI:** 10.1186/s12911-016-0304-5

**Published:** 2016-06-08

**Authors:** Sarah Bauerle Bass, Thomas F. Gordon, Ryan Gordon, Claudia Parvanta

**Affiliations:** Department of Social and Behavioral Sciences, Risk Communication Laboratory, Temple University, College of Public Health, 1301 Cecil B. Moore Ave., Philadelphia, PA 19122 USA; Department of Psychology, University of Massachusetts-Lowell, Health and Social Sciences Bldg., Suite 300, 113 Wilder St., Lowell, MA 01854 USA; Department of Behavioral and Social Sciences, University of the Sciences, 600 S. 43rd St., Philadelphia, PA 19104 USA

**Keywords:** Eye tracking, Low literacy, Risk communication, Emergency preparedness, “dirty bomb”, Decision-making

## Abstract

**Background:**

Eye tracking is commonly used in marketing to understand complex responses to materials, but has not been used to understand how low-literacy adults access health information or its relationship to decision making.

**Methods:**

This study assessed how participants use a literacy appropriate “dirty bomb” decision aid. Participants were randomized to receive a CDC “factsheet” (*n* = 21) or literacy-appropriate aid (*n* = 29) shown on a computer screen. Using 7 content similar slides, gaze patterns, mean pupil fixation time and mean overall time reading and looking at slides were compared. Groups were also compared by literacy level and effect on 'confidence of knowledge' and intended behavior.

**Results:**

Results revealed differing abilities to read densely written material. Intervention participants more precisely followed text on 4 of 7 content-similar slides compared to control participants whose gaze patterns indicated unread text, or repeated attempts at reading the same text, suggesting difficulty in understanding key preparedness messages. Controls had significantly longer pupil fixations on 5 of 7 slides and spent more overall time on every slide. In those with very low literacy, intervention participants were more likely than controls to say they understood what a “dirty bomb” is and how to respond if one should occur.

**Conclusions:**

Results indicate limited- literacy adults, especially those with very low literacy, may not be able to understand how to respond during a “dirty bomb” using available materials, making them vulnerable to negative health events. This study provides insights into how individuals perceive and process risk communication messages, illustrating a rich and nuanced understanding of the qualitative experience of a limited literacy population with written materials. It also demonstrates the feasibility of using these methods on a wider scale to develop more effective health and risk communication messages designed to increase knowledge of and compliance with general health guidelines, and enhance decision making. This has application for those with learning disabilities, those with limited media-literacy skills, and those needing to access the diverse array of assistive technologies now available. Eye tracking is thus a practical approach to understanding these diverse needs to ensure the development of cogent and salient communication.

**Electronic supplementary material:**

The online version of this article (doi:10.1186/s12911-016-0304-5) contains supplementary material, which is available to authorized users.

## Background

Eye tracking technology assesses how individuals view visual content by monitoring and recording eye movements as a subject processes the stimulus in real time. All systems use cameras, with some mounted to a flat stable surface and others embedded in a computer screen or mounted on lightweight glasses. Eye tracking is commonly used in marketing research [1], training systems involving hand-eye coordination (e.g., pilot flight training, surgery training) [2, 3], academic applications such as reading instruction or studies of cognition and information processing [4], and digital design work (e.g., websites, smart phones and tablet applications) [5, 6]. Eye movements, and the time spent on any one passage or image, indicate an individual’s interest or attention and provide an index of the input of information to more complex processing and/or reasoning [7, 8]. Eye tracking output can also clearly differentiate whether an individual is reading text as expected [9–12] or if a subject runs their eyes repeatedly over the same passages, suggesting difficulty in processing. The relationship between eye-tracking and comprehension has been studied since the early 1990’s, throughout which eye-tracking methodologies have made valuable contributions to our understanding of how to integrate information from texts, graphics and images to maximize comprehension. Current studies indicate brain structural correlates to text and sentence construction with comprehension can be validated with eye tracking methods [13]. Eye tracking can also be used to pinpoint specific pieces of content that pose comprehension difficulties, and these measures can be related to other outcome measures, such as knowledge gained, decision making, and intended or actual behavior. As such, eye movement measures can provide valuable information for the design or refinement of health communication materials, particularly when these combine language and visual information processing [14, 15], to enhance decision-making.

While eye tracking has been used in a number of health-related studies [15–19] few have used it to understand the needs of low-literacy groups [9, 20], and none have used eye tracking to test a decision aid for readability and comprehension by intended users with low literacy, comparing text that is written in an accessible format to text that is typical of the type of health information available to the public on the Internet. The present study fills this gap by testing a decision aid designed to provide information about what to do in the event of a “dirty bomb” explosion in an urban area.

The importance of examining low-literacy populations’ perceptions of and responses to Radiological Terror Events (RTEs) such as a “dirty bomb” is evident. The 2003 National Assessment of Adult Literacy, the most recent national-level assessment of literacy, revealed that 14 % of adults in the United States have below basic literacy, 22 % have basic literacy, 53 % have intermediate literacy, while only 12 % are proficient [21]. Overall, the United States ranks 17^th^ of 24 developed nations in literacy ability [22]. The *National Center for Education Statistics* (NCES) found that having only basic health literacy was greater in urban areas (16.3 %) compared to non-urban areas (13.1 %), with minorities and those over age 65 being most at risk of having literacy issues [23]. This prevalence has significant implications for communicating threats about terror, especially terror events that may be difficult to conceptualize, such as an explosion that includes radiation exposure.

The needs of low-literacy populations in the event of a terror or bioterror event are not well understood. This is especially critical because as we move farther away from the September 11, 2001 attacks, a decrease in resources directed toward emergency preparedness has occurred. RTEs, such as the explosion of a “dirty bomb”, present a threat that requires national, state, and local preparedness [24], but little research has been conducted on the public’s likely response. Studies that have been done indicate that potential threats from nuclear technologies and radiation contamination are increasing exponentially [25, 26], while at the same time these types of terror attacks are not well understood, and thus trigger exceptional feelings of helplessness, confusion, fear, and distress [27, 28].

An added area of concern is the public's trust of government authorities in times of crisis. While “sheltering in place” in an intact structure is the recommended way to stay safe in the event of a “dirty bomb” explosion, research shows that barriers to complying with government directives include distrust and lack of confidence in government or public health authorities, fear of subsequent attacks, and beliefs that needed supplies or help will not be made available [29–31]. These are serious factors that are likely to impede the acceptance of authority directives and decrease informed decision making. Several studies have noted a distrust of the government, particularly among minority populations and those with low education levels, due to the fear of discrimination or unequal treatment during such emergencies [30–32]. Most of these studies, however, did not focus on the unique challenges of those with low literacy, who may not only be less trusting of information, but also not able to access or understand information if presented. In a study that specifically examined how people would behave during RTEs, only 60 % of the participants reported that they would comply with instructions given by emergency preparedness planners [33]. In addition, little is currently known about the perceptions and understanding of low-literacy individuals concerning dirty bombs. A study conducted by Wray et al. found that participants preferred messaging that provided clear and accurate information and concrete action steps that were simple and consistently presented; since individuals were often confused by terms and messages utilized in current governmental communication efforts (e.g., “shelter in place”) [31]. Additionally, non-English speakers had difficulty understanding messages and feared they would miss critical information [31]. Currently, however, no studies have developed or tested literacy appropriate health communication materials or interventions for urban adults with limited literacy, but there is evidence from other domains, such as healthcare, that designing materials for literacy has effects not only on understanding of materials but on behavior as well [34–36].

The present study sought to address this gap by using eye tracking to assess how a systematically-developed, literacy appropriate decision aid, designed to foster understanding about how to respond to a “dirty bomb” event, addressed the needs of adults with limited literacy compared to a higher-literacy sample. The content of the decision aid was based on “Frequently Asked Questions” information developed by the Centers for Disease Control and Prevention (CDC), one of the few materials on RTEs publically available. Results showed clear patterns of differential text use in a small randomized pilot and demonstrated that eye tracking is a viable and useful tool in the quest to design materials for low-literacy groups to enhance decision-making.

## Methods

This study included a multi-phase protocol. Phase I included qualitative focus groups and quantitative surveys. Results of this phase were used in Phase II to develop a low-literacy risk communication decision aid about “dirty bomb” RTEs. Phase III involved testing the developed decision aid in a pilot randomized controlled trial. This paper discusses the results of Phase III, and focuses on the eye-tracking results.

### Study phases

***Phase I*** included both qualitative and quantitative data collection. This involved three focus groups with thirty-seven urban 18 to 65-year-olds, representing both men and women recruited from areas with low income and education levels in Philadelphia, PA [37]. From existing literature on perceptions of radiation and radiological terror, a moderator’s guide was developed and tailored for the target sample [38]. These questions helped to identify the range of cognitive and affective issues thought to be important for understanding perceptions of dirty bombs, including: (1) perceptions of emergency situations, (2) knowledge of a dirty bomb, (3) reaction to dirty bomb scenarios, and (4) comprehension of information provided. Results were analyzed using the Krueger method of analyzing narrative text, including familiarization, identifying a thematic framework, indexing, charting, mapping, and interpretation [39, 40].

Focus group results were then used to develop a comprehensive survey administered to 50 low-literacy adults, which assessed perception and understanding of a “dirty bomb,” trust of information sources, and intention to comply with government directives about “sheltering in place.” Implemented as a cross-sectional intercept survey, recruitment of respondents occurred in a variety of community-based sites in North Philadelphia, including local hospitals, neighborhood youth and senior community centers, and supermarkets. Four groupings of conceptually-related statements were included in the survey, including: 1. Statements on knowledge of a dirty bomb, trust of information sources if a dirty bomb occurred, and need for information about who was responsible for the event; 2. Statements on action intentions (staying home vs. leaving; getting children/family), belief in general preparedness, and preparedness activities such as having an emergency plan, food, and water; 3. Statements on what respondents would be worried about in the event of a dirty bomb, such as safety of food and water, risk of illness, breathing radiation, and its effects and risks to children and pets; and, 4. Statements that assessed participants’ perspectives of how likely a dirty bomb is to occur; likelihood of this event occurring compared to other threats (such as violence, flooding, or a car accident); beliefs about the trustworthiness of local, state, and federal authorities; and, whether their neighborhood would be treated fairly in response/recovery efforts.

***Phase II*** consisted of analyzing survey results using a variety of methods, including perceptual mapping and message vector modeling, and a cluster analysis to identify potential differences within the sample group that might need to be addressed in the design of the resulting decision aid [41]. Perceptual Mapping uses multidimensional scaling analysis to produce a three-dimensional graphic display of how participants perceive the relationships among the set of elements, by modeling the similarities and dissimilarities as distances between points in a multidimensional space. The resulting maps display the risk/benefit elements relative to each other and to “Self”, which can be an individual or group average. Perceptual mapping thus provides a graphical representation of how respondents conceptualize the decision or situation being evaluated. These methods are used extensively in marketing and advertising, and have been used to evaluate a number of public health decisions by the authors [42–45]. Message vector modeling can then be used to identify optimum message concept combinations for changing the positioning of elements in the mapping display (i.e. changing the perceptions or attitudes of the target segment). After the perceptual map is produced to display the relationships among concepts and between the concepts and “self”, vector modeling is used to determine which concept or concepts should be emphasized in a message or intervention to “move” the group toward the desired behavior, in this case to “shelter in place” in the event of a “dirty bomb”. This desired behavior establishes the target vector. (For more information on perceptual mapping and message vector modeling techniques, see http://sites.temple.edu/turiskcommlab/).

Based on these results, we developed a literacy-appropriate decision aid, optimizing the fit between the subjects’ perceptual maps and the content and format of literacy-appropriate information about a “dirty bomb”. The resulting decision aid included overall information about what a “dirty bomb” is and how to respond should an explosion occur, as well as specific message concepts determined to be important through the perceptual mapping. These concepts included: (1) Emphasizing trust in the information and recommended responses of authorities/experts, (2) Complying with recommended actions, and (3) Getting prepared for an emergency before one occurs. We also emphasized that in such a crisis, it is less important to dwell on who was responsible for the attack (a common need felt by victims of attacks that can impede taking immediate action) than it is to determine how to immediately protect yourself and your family. As well, the decision aid presented straight-forward information about what a dirty bomb is and how to respond if one occurred.

**Fig. 1:**
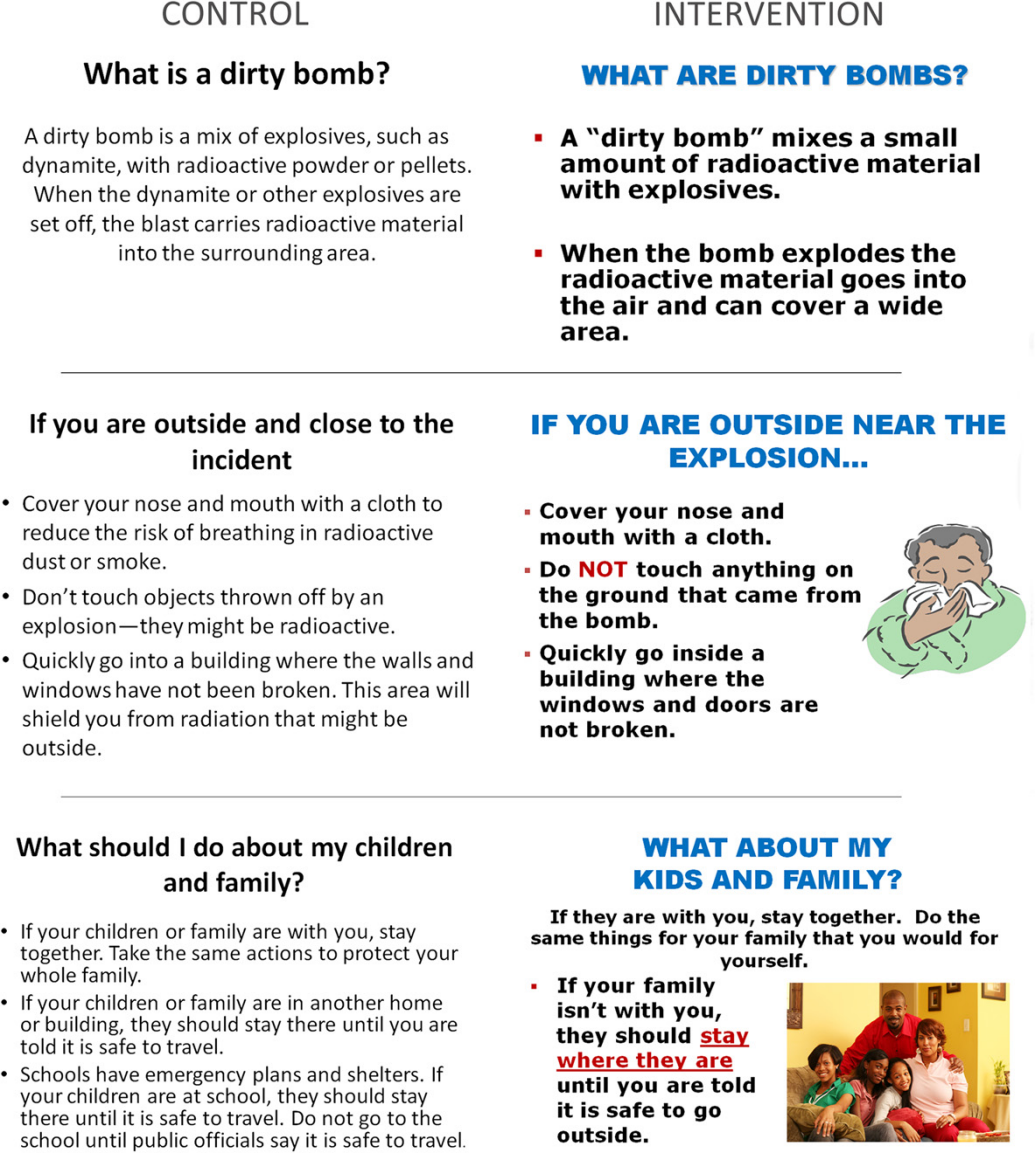
Dirty bomb decision aids

The control material was the CDC’s printed document, “Frequently Asked Questions” for a “dirty bomb”, which is available to the public on the CDC website (http://emergency.cdc.gov/radiation/dirtybombs.asp) and presented in a question/answer format. The reading level of this material, assessed using the Flesch-Kincaid test [46], was at an 8.2 grade level. Since the text on CDC’s website involved scrolling down a page and we wanted to be able to compare how participants read unique text and compare it across groups, both control and intervention materials were presented through a PowerPoint slide format, with each “question and answer” on a different slide. To focus on literacy relevant variables, the layout, graphics, and basic format of the low-literacy decision aid was made consistent, but text was written at a 5^th^ grade reading level and utilized graphics to represent words. The CDC FAQs decision aid was 19 slides, compared to the intervention (low-literacy) decision aid, which was 35 slides. Many slides were content similar, but the intervention decision aid was longer to account for special message content derived from the perceptual mapping but not addressed in the CDC's FAQs. Figure 1 illustrates differences between the Control decision aid (CDC's content) and the Intervention decision aid (designed for low-literacy individuals) on three content-similar slides.

During ***Phase III*** of the study, a randomized pilot-test comparing the developed literacy appropriate “dirty bomb” decision aid (intervention) and the existing CDC “dirty bomb” FAQs (control) was conducted with a convenience sample of 50 adults (29 in the intervention group, 21 in the control group) in the Risk Communication Laboratory at Temple University in Philadelphia. The testing occurred over six months, between January and June, 2012. We conducted this pilot study to assess the feasibility of conducting a fully powered randomized trial on whether a systematically developed, literacy appropriate decision aid would help urban residents understand the risks of a “dirty bomb” and how to respond if one occurred, as well as to determine if eye tracking was a useful and valid method of determining how those with low-literacy can or cannot access text.

Participants were recruited for the pilot through community-based methods, including placing flyers in the Temple University Hospital’s General Internal Medicine Clinic, community organizations surrounding Temple University, as well as local pharmacies and supermarkets. Flyers included a “tear tab” with a phone number so those interested could call about participating in the study. We also focused on users of a neighborhood food bank, a senior services organization, and a federal services office. In these cases, research assistants visited the sites, talked with people about the study and screened them for eligibility. The goal was to have participants who closely represented people who matched the socio-demographic groups represented in the focus group and survey analysis.

Screening included demographic information (age, gender, ethnicity and education level) and health literacy level. In-person literacy screening utilized the Rapid Estimate of Adult Literacy in Medicine – Revised (REALM-R) test, which is a word recognition test in which participants are asked to pronounce eleven medical words of increasing difficulty [47]. The first three words are not scored, giving the test a scored range of 0–8. Participants unable to pronounce the first three words have very low literacy skills and those unable to pronounce more than six are at risk of having inadequate literacy. In this study, those scoring a 6 or below were eligible. Over the phone literacy screening utilized the Single Item Literacy Screening tool (SILS) [48] and participants qualified if they indicated they always, often or sometimes “need help reading instructions, pamphlets or other written material from a doctor or pharmacy”. Other eligibility criteria included being 18 or over, being able to read and speak English, and the absence of dyslexia or other learning problems that interfere with reading/tracking text. If eligible, appointments to come into the Risk Communication Laboratory were made and participants were randomized to the control and intervention groups using a random numbers table. Participants were provided incentives to take part in the study, including a $20 gift card and two transit tokens. The university's Institutional Review Board approved all procedures and consent materials.

When participants arrived at the Risk Communication Laboratory a trained research assistant orally administered all consent forms and instruments, which were tailored for people with low literacy. The eye tracking data collection procedures were explained to participants using pictures of the equipment and samples. Participants were then escorted to the testing lab, which is a small private room, and they were connected to the eye tracking system in preparation for viewing their assigned decision aid. The input from the physiological monitors was fed directly into a computer database for storage and analysis. Eye tracking was performed using an Applied Science Laboratory stationary eye tracker (Eye Trac 6000). This equipment requires a participant to sit in front of a screen with a Velcro band around his/her head, which automatically corrects for any head movement during eye tracking data collection. The control and intervention messages were presented on a computer monitor for each participant to view. Once the system acquired a pupil lock and calibrated the subject’s eye tracking, he/she was told to look at the screen and read the slide presented, and then indicate to the technician when he/she was done. The technician then advanced the slide for the participant until he/she was done looking at all slides. Once the participant was finished, he/she was directed back to another room where the post-education survey protocol was conducted. The full study took approximately 45 min to administer. Results of eye tracking analyses are discussed here.

### Analysis

The two groups (Intervention v. Control) are profiled in Table 1 on demographic factors. In addition, all participants were English speaking, with none reporting English as a second language. While all participants had low literacy, we also dichotomized this variable to assess whether differences were seen in those with very low literacy (defined as those who scored a 0–2 on the REALM-R or said they “always” or “frequently” have trouble filling out medical forms on the SILS), compared to those with low literacy (defined as those who scored a 3–6 on the REALM-R or said they “sometimes” have trouble filling out medical forms of the SILS). Demographic characteristics of these groups are provided in Table 2. For continuous variables (Tables 3, 4 and 5), means, sample sizes and standard deviations are provided. Using SPSS Version 23, T-Tests were computed to examine differences between groups as well as sub-groups by literacy level. Four groups were used: 1. Intervention, Very Low Literacy; 2. Intervention, Low literacy; 3. Control, Very Low Literacy; and, 4. Control, Low Literacy. Group differences were examined on mean qualitative ratings of ability to track text, average pupil fixation, and average time to complete reading a slide, a composite average of these ratings, as well as outcome measures of confidence in knowledge of what a “dirty bomb” is and intended behavior in the likelihood of a “dirty bomb” exploding.

**Table 1:** Demographic Characteristics and Literacy Test Means, RCT Intervention and Control Groups

	Number		Percent	
	Control	Intervention	Control	Intervention
Age				
23–35	4	3	19.05	11.11
36–50	9	18	42.86	66.67
51–59	7	5	33.33	18.52
60–67	1	1	4.76	3.70
Total	21	27	100.00	100.00
Race				
African American	20	24	95.24	82.76
Mixed	0	1	0.00	3.45
White	1	2	4.76	6.89
Asian	0	1	0.00	3.45
Native American	0	1	0.00	3.45
Total	21	29	100.00	100.00
Hispanic Origin				
Yes	0	2	0.00	7.14
No	20	26	100.00	92.86
Total	20	28	100.00	100.00
Gender				
Male	10	16	47.62	55.17
Female	11	13	52.38	44.83
Total	21	29	100.00	100.00
Education				
Grade School	0	2	0.00	7.14
Some High School	7	7	35.00	25.00
Graduated High School	11	15	55.00	53.57
GED	1	2	5.00	7.14
Some College	1	1	5.00	3.57
Graduated from College	0	1	0.00	3.57
Total	20	28	100.00	100.00
Literacy Scores				
REALM-R (mean)	3.00 (*N* = 11)	2.35 (*N* = 20)		
SILS (mean)	3.27 (*N* = 8)	3.50 (*N* = 11)

**Table 2:** Participant Demographic Characteristics by Literacy Level

	Literacy level	
	Very low literacy	Low literacy
Gender (*n* = 50)		
Male	15 (30 %)	11 (22 %)
Female	14 (28 %)	10 (20 %)
Race (*n* = 50)		
African American	25 (50 %)	19 (38 %)
Mixed	1 (2 %)	0
White	1 (2 %)	2 (4 %)
Asian	1 (2 %)	0
Native American	1 (2 %)	0
Education Level (*n* = 48)		
Grade school	2 (4.2 %)	0
Some High School	10 (20.8 %)	4 (8.3 %)
HS Graduate/GED	13 (27.1 %)	16 (33.3 %)
Some College	1 (2.1 %)	1 (2.1 %)
Graduated College	1 (2.1 %)	0
Age (*n* = 48)		
23–35	6 (12.5 %)	1 (2.1 %)
36–45	6 (12.5 %)	7 (14.6 %)
46–55	15 (31.3 %)	10 (20.8 %)
55+	0	3 (6.3 %)

**Table 3:** Independent Samples t-Tests: Eye Tracking Ratings on Seven Selected Slides

	Intervention Group						95 % CI for Mean Difference		
	Intervention			Control					
Slide	M	SD	n	M	SD	n		t	df
1. What is a dirty bomb?	3.53	1.13	17	4.00	1.00	11	−.387,1.33	1.13	26
2. A dirty bomb is not an atomic bomb.	3.65	1.49	17	2.50	1.29	14	−2.19,−.11	2.26*	29
3. What to do if you’re outside	3.81	1.17	16	2.75	1.06	12	−1.94,−.18	2.48*	26
4. What to do if you’re inside.	3.88	.96	16	2.31	1.38	13	−2.46,−.68	3.61**	27
5. What should I do about my children and family?	3.53	1.06	15	2.38	1.19	13	−2.02.−.27	2.70*	26
6. What to do about food and water?	3.41	1.29	17	2.60	1.18	15	−1.71,.08	1.86	30
7. How do I know about radiation exposure?	3.41	1.33	17	2.64	1.22	14	−1.71,.17	1.67	29

Scale = 5 points. Larger means more consistency/precision of eye-tracking

*p ≤ .05, **p ≤ .005

**Table 4:** Independent Samples T-Tests: Number of Pupil Fixations by Seven Selected Slides

	Intervention Group						95 % CI for Mean Difference		
	Intervention			Control					
Slide	M	SD	n	M	SD	n		t	df
1. What is a dirty bomb?	24.00	20.69	24	23.67	22.29	21	−13.26,12.59	.05	43
2. A dirty bomb is not an atomic bomb.	15.33	15.02	24	38.14	30.05	21	8.80,36.82	3.28**	43
3. What to do if you’re outside	32.46	32.62	24	35.95	36.67	21	−17.33,24.32	.34	43
4. What to do if you’re inside.	28.13	28.13	24	47.14	38.22	21	.15,37.89	2.03*	43
5. What should I do about my children and family?	32.04	32.04	23	50.00	36.46	21	−1.48,37,40	1.86	42
6. What to do about food and water?	36.71	36.91	23	50.52	37.54	21	−6.58,33.79	1.36	42
7. How do I know about radiation exposure?	24.96	24.96	23	54.20	43.89	20	4.71,47.60	2.46*	42

*p ≤ .05, **p ≤ .005

**Table 5:** Independent Samples T-Tests: Time Spent Reading the Seven Selected Slides

	Intervention Group						95 % CI for Mean Difference		
	Intervention			Control					
Slide	M	SD	n	M	SD	n		t	df
1. What is a dirty bomb?	12.66	4.53	24	21.94	17.32	21	1.89,16.68	2.54*	43
2. A dirty bomb is not an atomic bomb.	9.54	8.90	24	32.35	16.81	21	14.87,30.76	5.79***	43
3. What to do if you’re outside	17.37	9.22	24	27.29	17.16	21	1.79,18.06	2.46**	43
4. What to do if you’re inside.	16.70	6.88	24	31.61	24.02	21	4.58,25.23	2.91**	43
5. What should I do about my children and family?	17.28	7.35	23	35.72.	24.06	21	7.83,29.06	3.51**	42
6. What to do about food and water?	17.92	7.27	23	34.85	14.30	21	10.12,23.75	5.02***	42
7. How do I know about radiation exposure?	12.25	5.99	23	31.57	32.05	20	5.58,33.06	2.84**	41

*p ≤ .05, **p ≤ .005, ***p ≤ .000

To extract pupil fixation and average time to complete a slide, as well as extracting the gaze patterns, the Eyenal software program (designed for analysis of eye-tracking data) was used (Applied Science Labs). In addition, a 5-point subjective rating scale was developed and tested by the authors to rate the consistency of a participant's gaze patterns, including the ease with which the participant appeared to follow the text. To establish inter-coder reliability in the use of this scale, three study coders first came to agreement about what each scale point meant. The five-point tracking consistency scale was defined as follows:

1. =“Very chaotic tracking with very limited ability to follow text”,
2. = "Mostly chaotic tracking and quite limited ability to follow text,"
3. = “Mixed tracking, where tracking of some areas of the text/slide were precise, and the rest chaotic and inconsistent,"
4. = "Mostly precise tracking and relatively consistent following of text,"
5. = "Very precise tracking and highly consistent following of text."

Coder training involved practicing use of the scale to rate eye-tracking consistency until a high level of inter-coder reliability was achieved (Pearson r = .898, Spearman R = .989). After training, coders independently rated each participant's eye-tracking patterns on each slide. In the few cases where rater discrepancies occurred, the coders reviewed the gaze pattern in question together, and discussed their ratings to arrive at a consensus scale value.

Eye-tracking comparisons were only performed on seven content-similar slides to control for content. These seven slides included similar questions and responses and allowed us to compare eye tracking patterns across similar content. The slides included:

1. What is a dirty bomb?
2. Dirty bomb is not the same as an atomic bomb.
3. What to do if you are inside and close to the explosion?
4. What to do if you are outside and close to the explosion?
5. What should I do about my kids and family?
6. Will my food and water be safe?
7. How do I know if I’ve been exposed to radiation?

## Results

As illustrated in Table 1, there were 29 participants in the intervention group and 21 in the control group. No significant differences between the groups were found on demographic variables and literacy levels, indicating randomization was successful. Sub-analysis of the group by literacy level (Table 2) showed similar characteristics in those who had very low literacy compared with those with low literacy. No significant differences by literacy group were found. For all groups, the dominant age category was 36 to 59 and they were primarily African American. The majority also reported graduating from high school. Literacy statistics indicated a very low level of literacy. For the 31 participants who did the REALM-R screening, the mean score was 2.58, with a range of 0 to 5 (SD 1.42) indicating very low literacy. Of the 19 participants screened over the phone with the SILS, the mean score was a 3.37 (range 3 to 5, SD = .684).

Table 3 summarizes independent- samples t-Tests, comparing the intervention and control groups across the seven content similar slides on gaze pattern and consistency of eye tracking. The intervention group was rated significantly higher in ability track on four of the seven slides, including the “A dirty bomb is not an atomic bomb” (M = 3.65, SD = 1.49; t(29) = 2.26, *p* = .032), “what to do if you are outside and close to the explosion” (M = 3.81, SD = 1.17; t(26) = 2.48, *p* = .02), “what to do if you are inside and close to the explosion” (M = 3.88, SD = .96; t(27) = 3.61, *p* = .001), and “what should I do about my children and family” (M = 3.53, SD = 1.06; t(26) = 2.70, *p* = .012) slides. Intervention participants had higher means on all but one of the slides (“What is a dirty bomb”), which in both groups was very simple and contained only one statement describing what a dirty bomb is.

**Fig. 2:**
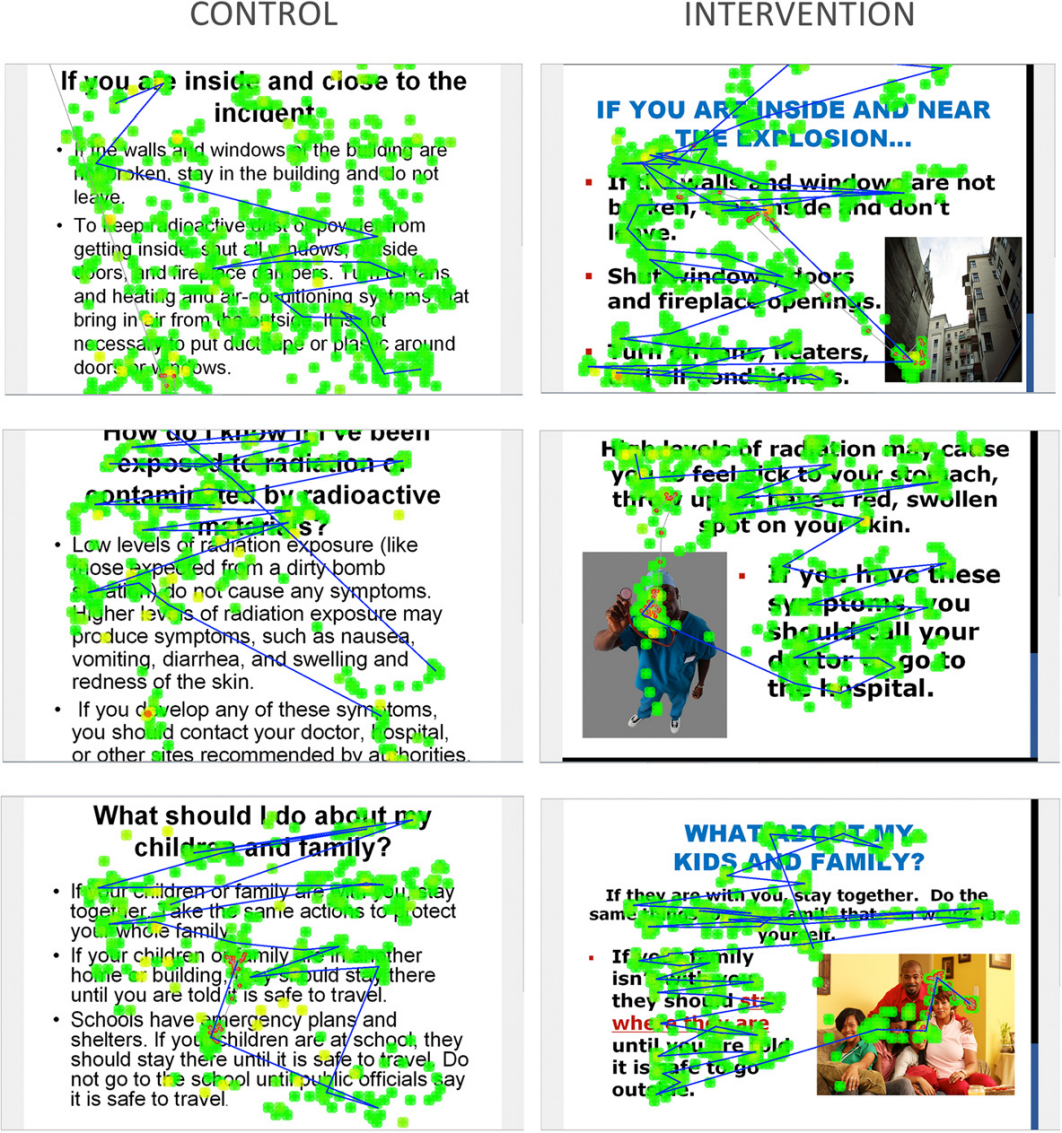
Sample eye tracking – various slides

Figure 2 shows summary slides of selected participant eye tracking to illustrate the differences evident in tracking patterns; each represents a different participant. The blue line indicates the actual pattern of tracking and the green dots indicate pupil fixation (the larger the dot, the longer the participant spent looking at that location). These patterns illustrate the differences observed between participants presented with higher vs. lower literacy information. For example, in Slide 1 – Control, the participant looked all over the page but did not have a specific gaze pattern that indicated he/she was actually reading the text. Slide 2 – Intervention, however, shows a clear gaze pattern indicating the participant was following the text as would be expected if he/she was reading the text. It is also clear that he/she looked at the photograph on the slide after reading the text. Similarly, in slides 3 and 5 – control, it appears that the participants made an attempt to read the title and the first sentence of text but then did not finish, not looking at the rest of the text. Slides 4 and 6 – intervention, however, clearly show the participants not only followed the text, but also spent time looking at the photographs. (Additional files 1 and 2: Videos to Fig. 2 are provided, which show real-time eye tracking for selected intervention and control participants.)

Mean number of pupil fixations on the seven selected slides indicated that the control group spent significantly longer looking at specific words or phrases on three of the slides (Table 4). These include “A dirty bomb is not an atomic bomb” (M = 38.14, SD = 30.05; t(43) = 3.28, *p* = .002), “What to do if you’re inside” (M = 47.14, SD = 38.22; t(43) = 2.03, *p* = .048), “How do I know about radiation exposure” (M = 54.20, SD = 43.89; t(42) = 2.46, *p* = .02). A fourth slide, “What should I do about my children and family” approaches significance, with the control group having more mean fixations (M = 50, SD = 36.46; t(42) = 1.86, *p* = .069). This is indicative of the more densely worded material in the control slides and their low-literacy level. In some cases, control participants had twice the number of pupil fixations compared to the intervention participants, most likely because of the density of the text. This correlates with the observations of real time tracking, where control participants can be seen repeating lines of text or skipping back and forth on the slide.

Table 5 illustrates the mean time it took participants to read/look at each slide. Participants were allowed to take as much time as they wanted on each slide and were told to tell the technician when they were done and were ready to move to the next. As illustrated, control participants took significantly longer to read all seven compared slides, sometimes taking twice as long as intervention participants. Even on the simple “What is a dirty bomb” slide, control participants spent on average 21.94 s (SD = 17.32) looking at the slide compared to just 12.66 s (SD = 4.53) spent by intervention participants (t(43) = 2.54, *p* = .015). Similar patterns are seen in the other slides: “An dirty bomb is not an atomic bomb” (M = 32.35 s, SD = 16.81; t(43) = 5.79, *p* = .000), “What to do if you’re outside” (M = 27.29 s, SD = 17.16; t(43) = 2.46, *p* = .018), “What to do if you’re inside” (M = 31.61 s, SD = 24.02; t(43) = 2.91, *p* = .006), “What should I do about my children and family” (M = 35.72 s, SD = 24.06; t(42) = 3.51, *p* = .001), “What to do about food and water” (M = 34.85 s, SD = 14.30; t(42) = 5.02, *p* = .000), and “How do I know about radiation exposure” (M = 31.57 s, SD = 32.05; t(41) = 2.84, *p* = .007). This correlates with both the eye tracking ratings and the pupil fixation analysis and strengthens the argument that the control participants had a difficult time reading and processing the text-heavy control slides.

In addition to looking at individual slides, composite scores for overall ability to track slides, fixations and time were calculated and then correlated to the dichotomized literacy variable. Pearson Correlations indicates that literacy level was associated with four of the 7 slides on the eye tracking rating (Dirty Bomb is not an Atomic Bomb, .404, *p* = .024; What do with family, .409, *p* = .031; What to do about food/water, .473, *p* = .006; Radiation Exposure, .406, *p* = .024). In addition, comparing average time, fixation and tracking ratings by intervention group, the Intervention, Very Low Literacy group had lower time and fixation composite scores (time = 32.08 s vs. 40.20 for Control, Very Low Literacy; fixation = 39.97 vs.49.37) and higher tracking scores (3.32 vs. 2.79). These differences, however, were not significant.

Finally, Independent Samples T-Tests were run comparing effects of the intervention on those with the lowest literacy levels on outcome measures, specifically confidence in knowledge of what a “dirty bomb” is, and intended behavior (i.e. sheltering in place) if one occurred (participants indicated how much they agreed or disagreed with a statement on a 0-10 scale). Table 6 illustrates that significant differences were seen in three post-intervention knowledge or behavior survey questions: Know what a dirty bomb is (M = 8.76, SD = 2.64; t(28) = 2.78, *p* = .01); Know what to do if a bomb blows up (M = 8.95, SD = 1.50; t(28) = 2.49, *p* = .02); My ability to follow instructions if a “dirty bomb” happens (M = 8.80, SD = .62; t(27) = 2.87, *p* = .008). These differences were not seen in the Low Literacy by intervention groups.

**Table 6:** T-Tests, Very Low literacy by Intervention Group on Knowledge and Behavior Post-Intervention Survey Questions

	Intervention Group						95 % CI for Mean Difference		
	Intervention			Control					
Question	M	SD	n	M	SD	n		t	df
1. I know what a dirty bomb is.	8.76	2.64	21	5.44	3.75	9	.87, 5.77	2.78*	28
2. I know what to do if a bomb blows up.	8.95	1.50	21	6.67	3.61	9	.40, 4.17	2.49*	28
3. How do you feel about your ability to follow instructions after a dirty bomb?	8.80	.62	20	7.44	1.94	9	.386, 2.3	2.87*	27

Scale was 0–10, with zero = totally disagree and 10 = totally agree

*p ≤ .05

## Discussion

Monitoring of eye tracking in real time and watching video of each participant’s session clearly revealed that in this pilot study the control group participants had a difficult time getting through the dense text. For many of the low-literacy participants, it was obvious that they either chose to randomly glance around the slide text and not systematically work through it, or they began to work through the text, often re-reading given lines several times before giving up and resorting to random looking patterns. In some instances, it seemed that control participants would look off the slide for a period of time before deciding enough time had passed to tell the technician to advance to the next slide. Observing these patterns could be difficult to watch, as the apparent frustration many participants had with the text was evident. These observations were supported by the data, which indicated significant differences in not only the gaze patterns tracked across time, but was also in the measures of time spent at each fixation point, and the total number of fixation points per slide. These differences also remained once tracking fixations and time were combined as a composite score and compared, and when the sample was compared by their literacy level and intervention group. It was clear that the dense text provided in the control condition caused participants to spend more time with trying to get through the text and attempting to read lines more than once. Despite the small sample size of this study, the differences we found in tracking and outcome measures (confidence in knowledge and ability to follow instructions) indicate a large effect size (cohen’s d = .94). However, future studies using eye tracking measures will have to take into account the intent of the trial (eye tracking as outcomes vs. changes in knowledge and behavior) and whether larger sample sizes are required to test the effect of these types of materials are larger audiences.

Clearly, having access to literacy appropriate materials is important. In this study, significant effects on confidence in knowing what a dirty bomb was, how to respond if one occurred and ability to carry out instructions was seen in those with the very lowest literacy. This is a significant finding, indicating that even those with very low literacy can benefit from having information presented in a manner that addresses their needs. Because of the serious consequences of exposure to a “dirty bomb’, it is important that disaster planning and management teams address the accessibility of risk communication materials designed for limited-literacy groups, so that everyone can have equal access to potentially lifesaving information and make informed decisions for themselves and their families. Health literacy studies suggest that a third-to-a-half of the US population has some difficulty using written materials [49], such as those available on the CDC website -- materials that have been measured at an 8^th^ grade reading level or higher [50, 51]. If large numbers of US adults cannot use complex prose, it is important public health communication specialists develop materials that are accessible and presented in a developmentally appropriate form. If these materials are not available, people with limited-literacy will be unable to adequately prepare for or respond to an emergency such as a “dirty bomb”, thus decreasing their ability to make informed decisions.

Public trust and confidence can help mitigate and improve the ability to manage a terror threat [52], but this is severely compromised if a large group is unable to access or process emergency communications. Risk communication is essential to containing the public’s fear and fatalism, and for ensuring public cooperation during crisis events [53]. However, risk communication is especially challenging when applying the messages to high risk, high outrage situations [54] such as a “dirty bomb” due to their capacity to produce widespread fear, which reduces complexity of information processing and inhibits memory abilities [26]. These limitations are further accentuated in low literacy populations, as distrust may be exacerbated by use of complicated terminology that assumes the public has more information and understanding than they do and making them most at risk for negative outcomes.

On a conceptual level, the goal of effectively communicating a health/risk message to a particular target audience must take into account not only their literacy skills, but also their level of education and experience with the topic being addressed. As such, the design of effective messages strategies requires attention to the task of informing and educating the receiver of the message, and the task of creating an information processing experience that effectively and efficiently optimizes the formatting, graphics, and visual/auditory aspects of message structure. These latter structural elements are critical to optimizing overall processing, learning, motivation, decision making, and behavior. Eye tracking analyses are particularly well suited to understand both the content/educational aspects of message design and the structural information processing aspects, and to understand how difficult it may be to access material that is not developed for those with low literacy. In this pilot study it was clear that low-literacy participants were more comfortable with the decision aid written at their literacy level and were more receptive to its messages; this is especially true in those with the lowest literacy. Eye tracking was thus a viable and useful method for understanding how low literacy adults may respond to and understand emergency communications, informing the development of better, more accessible materials.

### Limitations and future research

The limitations of this research include the fact that as a pilot study the sample size is, by design, small. Although this is the case, care was taken to accurately assess the participant's literacy level and to randomly assign participants to the control and intervention test-groups. Future studies should expand the sample size and also expand the geographic scope of the study to explore urban v. suburban differences as well as potential regional differences across the country.

There are also other types of eye tracking data that could have been used in the analysis of this study, including saccades and smooth pursuits. These could be useful in larger studies to assess ease of tracking and reading material, especially when video or other interactive activities are incorporated into learning materials. These were not used here. In addition, the subjective eye tracking rating, despite rigorous training of raters and good reliability, is still a source of bias. Perhaps in a larger study this could be compared through a more objective mathematical evaluation of areas of interest and/or machine learning techniques. These measures were not available for this study. In addition, screening participants for visual acuity and vision quality would be advised.

Another limitation involves the current focus on the topic of radiological terror events (Dirty Bomb). Individual responses, in terms of information processing, physiological reactions, and knowledge gain will likely vary with different types of content (e.g., what to do in natural v. man-made disasters; active-shooter v. kidnapping/ransom events; bombs at a public event v. bombing a building, etc.). Public Health officials will have to develop "preparedness" materials for these types of differences as the frequency of such events continues to increase. This may also affect eye tracking results, as information more familiar to an audience may hinder the ability to differentiate as easily between tested materials.

A third important limitation of the present study is that it focuses on a particular aspect (literacy) of the audience's ability to access, interpret, and apply critical information about what to do in a particular emergency situation. The eye tracking and message design methodology used in this research can be directly applied to areas other than audience literacy levels. For example, it can be used to design more effective materials for individuals with learning disabilities, or those with limited media-literacy skills, or those needing to access the diverse array of assistive technologies now available.

## Conclusion

The use of eye tracking is an effective approach to understand how health communication materials are accessed and processed by individuals with limited-literacy skills. We believe that this pilot work clearly demonstrates the feasibility of using these methods on a wider scale to develop more effective health and risk communication messages designed to increase knowledge of and compliance with general health guidelines on the one hand and specific health-based emergency directives on the other. It is also clear that the message design and testing approach has application not only for those with low-literacy, but for those with learning disabilities, those with limited media-literacy skills, and those needing to access the diverse array of assistive technologies now available. The long-term goal is to evolve and refine powerful message design tools that will contribute to effective decision making and the elimination of health disparities evident in educational, social, and personal contexts.

## Abbreviations

CDC, Centers for Disease Control and Prevention; REALM-R, Rapid Estimate of Adult Literacy in Medicine-Revised; RTE, Radiological Terror Event; SILS, Single Item Literacy Screener

## Additional files

Additional file 1: a. Eye tracking video of Intervention condition – what about my kids and family. b. Eye tracking video of Control condition – what about my kids and family. (ZIP 32338 kb) Additional file 2: a. Eye tracking video of Intervention condition – what about my food. b. Eye tracking video of Control condition – what about my food. (ZIP 48292 kb)
